# An outbreak of tuberculosis in a middle school in Henan, China: Epidemiology and risk factors

**DOI:** 10.1371/journal.pone.0225042

**Published:** 2019-11-15

**Authors:** Jiying Xu, Guojie Wang, Yanqiu Zhang, Guolong Zhang, Jin Xing, Lihong Qi, Yan Zhuang, Hejun Zeng, Jianhua Chang

**Affiliations:** 1 Institute of Tuberculosis Control and Prevention, Henan Provincial Center for Disease Control and Prevention, Zhengzhou, Henan province, China; 2 Department of Medicine, Henan Medical College, Zhengzhou, Henan province, China; 3 Department of Public Health Science, School of Medicine, University of California, Davis, California, United States of America; 4 Institute of Tuberculosis Control and Prevention, Hebi Prefecture Center for Disease Control and Prevention, Hebi, China; Chinese Academy of Medical Sciences and Peking Union Medical College, CHINA

## Abstract

**Background:**

In 2013, a tuberculosis (TB) outbreak occurred in a middle school in Henan province of China. An outbreak survey was carried out in the school.

**Objectives:**

This study was undertaken to investigate the detection rate of TB cases and those with strong Mantoux positive (SMP), defined as tuberculin skin test (TST) indurations of 15mm or larger and/or blisters, necrosis or lymphangitis, and to identify their risk factors.

**Methods:**

The TST, chest x-ray/radiography, and TB-suspicious symptoms interview were used to screen for TB cases. Their diagnosis was made by sputum smear microscopy, liquid culture, computed tomography (CT), and diagnostic therapy if necessary. We retrospectively analyzed the outbreak survey data of 4082 students and 278 staff in the school. Logistic regression models were used to identify the risk factors associated with SMP and TB disease.

**Results:**

Approximately 3.55% of students and 16.55% of staff were SMP. SMP rate in students was significantly lower than that in staff (p<0.001). 55 TB cases in students and none in staff were identified in the school from February to November, 2013, with a detection rate of 1.35% for students. SMP and TB case detection rates were 20.29% and 41.77%, respectively, in the index class, both significantly higher than that in the other classes (3.26% and 0.55%, respectively; both p<0.001). In the index class, TB case detection rate over the study period in students with SMP was not significantly higher than that with TST indurations of <15mm (38.18% vs 21.43%, p = 0.24), but it was significantly higher in the other classes (7.81% vs 0.18%, p<0.001). Risk factors independently associated with SMP and TB cases shared the following with the index case: same dormitory-floor (adjusted odds ratio (AOR) 5.36, 95% CI 3.02–9.48, p<0.001 for SMP; AOR 2.67; 95% CI 1.03–6.96, p = 0.044 for TB cases), same classroom (AOR 4.01; 95% CI 1.95–8.26, p<0.001 for SMP; AOR 165.08; 95% CI 66.88–407.47, p<0.001 for TB cases), same teaching-floor in different classroom (AOR 5.41; 95% CI 3.02–9.71, p<0.001 for SMP; AOR 11.24; 95% CI 3.71–34.03, p<0.001 for TB cases) and same teaching-building on different floor (AOR 1.81; 95% CI 1.16–2.85, p = 0.009 for SMP; AOR 3.30; 95% CI 1.16–9.42, p = 0.025 for TB cases). The closer the contact was with the index case, the larger the AORs for SMP and TB cases were.

**Conclusion:**

Same dormitory-floor, same classroom, same teaching-floor and same teaching-building with the index case were all risk factors for both TB infection and disease for students in the outbreak, and the closer the contact was with the index case, the higher the risk was observed. Attention should also be paid to students with TST indurations <15mm, as well as to those with that ≥15mm for the index class in dealing with the outbreak.

## Introduction

Tuberculosis (TB) is one of the top 10 causes of death and the leading cause from a single infectious agent (above HIV/AIDS), worldwide. Millions of people continue to fall sick with TB each year. China is one of the 30 high burden countries of TB and ranks second in terms of the number of TB cases, with an estimated of 889,000 incident cases in 2017 [1]. Out of the 990,000 pulmonary TB cases registered in the National Disease Surveillance Information Management System in China in 2010, about 4.8% were students [2]. And a total of 48,289 student with pulmonary TB were reported in China in 2018, with the reported incidence of 17.97/100 000 [3]. Students rank second, next to farmers, in terms of TB ratio by occupation in Henan province [4]. TB could easily spread among students and lead to an outbreak once there is an infectious pulmonary TB case in a school, due to the highly crowded environment in school settings and the relatively weaker immune system in students. This is particularly true for boarding schools, where usually several students live in one room [5, 6]. Contact screening for latent TB infection and TB cases in schools is obligatory once there is a TB case in students or teachers reported according to related norm and manual in China [6].

On February 17, 2013, a student in a Middle School in Hebi city, Henan province (located in the middle of China with a population of 94 million and 64,236 pulmonary TB cases registered in 2013), was diagnosed as a sputum smear positive pulmonary TB case. A series of screenings and an outbreak survey were carried out in the school. In total, 55 students were diagnosed as TB cases from February to November 2013. To learn the association between TB cases, TB infection and sex, class, accommodation, and to identify factors related to TB disease detection and SMP in school settings, and ultimately, improving TB control in schools, we retrospectively analyzed the outbreak survey data.

There were 4102 students (2340 were boarding) in 60 classes (28 junior, 32 senior) and 289 staff (including temporaries) in the school. Five four-story teaching buildings were located in the campus with three classrooms on each story in most of buildings. Each classroom had an area of approximately 65 square meters. There were two five-story dormitory buildings for male and female students, respectively. Each dormitory housed ten students in an area of approximately 20 square meters. In addition, there was a two-story dining hall for students. There were 79 students in the index class, with 61 females and 18 males. Out of the 61 girls, 40 housed in four neighbor dormitories, four in other dormitories on different floor and the other 17 were non-boarders. For the 18 boys, 13 lived in two dormitories on the same floor and the other 5 were non-boarders.

## Methods

### Study design

We retrospectively reviewed the outbreak survey data, investigation records and medical records, including general information of students and the school, such as gender, classroom and residential status, distributions of classroom and dormitory, results of tuberculin skin test (TST) and clinical information of TB cases, to identify factors related to TB disease and strong Mantoux positive (SMP) in the school, and to analyze TB disease detection rate in different indurations of TST. We checked information with related staff in Hebi Center for Disease Control and Prevention (CDC), Hebi Contagious Diseases Hospital and the school when necessary.

### Case definition

Cases were defined according to the Guidelines for Implementation of National TB Control Program in China [7]. A laboratory-confirmed case was defined as two or more positive sputum smears (SS), or 1 positive SS plus a chest radiograph indicative of TB, or 1 positive SS plus 1 positive sputum culture (SC), or 1 positive SC plus 3 negative SS and a chest radiograph indicative of TB. A clinically diagnosed case was defined as a chest radiograph indicative of TB with 3 negative SS plus any one of the following: 1) a productive cough/hemoptysis lasting for at least 2 weeks; 2) a TST induration with a diameter of 15mm or larger and /or blisters, necrosis or lymphangitis (SMP); 3) excluding other lung diseases after a diagnostic therapy.

Classification of TB was according to the Hygiene Professional standards, Peoples Republic of China [8], and the Guidelines for Implementation of the National TB Control Program in China [7].

The index case was considered as the first smear positive patient diagnosed in the school[9]. The class with the index case was named as index class and the grade with the index case as index grade.

### Tuberculin skin test (TST)

TST was carried out and indurations measured at 48 to 72 hours by trained nurses from Hebi Contagious Disease Hospital. Purified protein derivative (PPD) produced from tubercle bacilli (TB-PPD, Beijing Sanroad Biological Products Co., Ltd., Beijing, China) was used for the third screening, and PPD produced from bacilli Calmette-Guérin (BCG-PPD, Chengdu Institute of Biological Products Co., Ltd., Chengdu, China) for the fourth one, with standard operating procedures. A strong Mantoux positive (SMP) was defined as TST indurations with a diameter of 15mm or larger and /or blisters, necrosis and lymphangitis [7].

### Case finding

**Fig 1:**
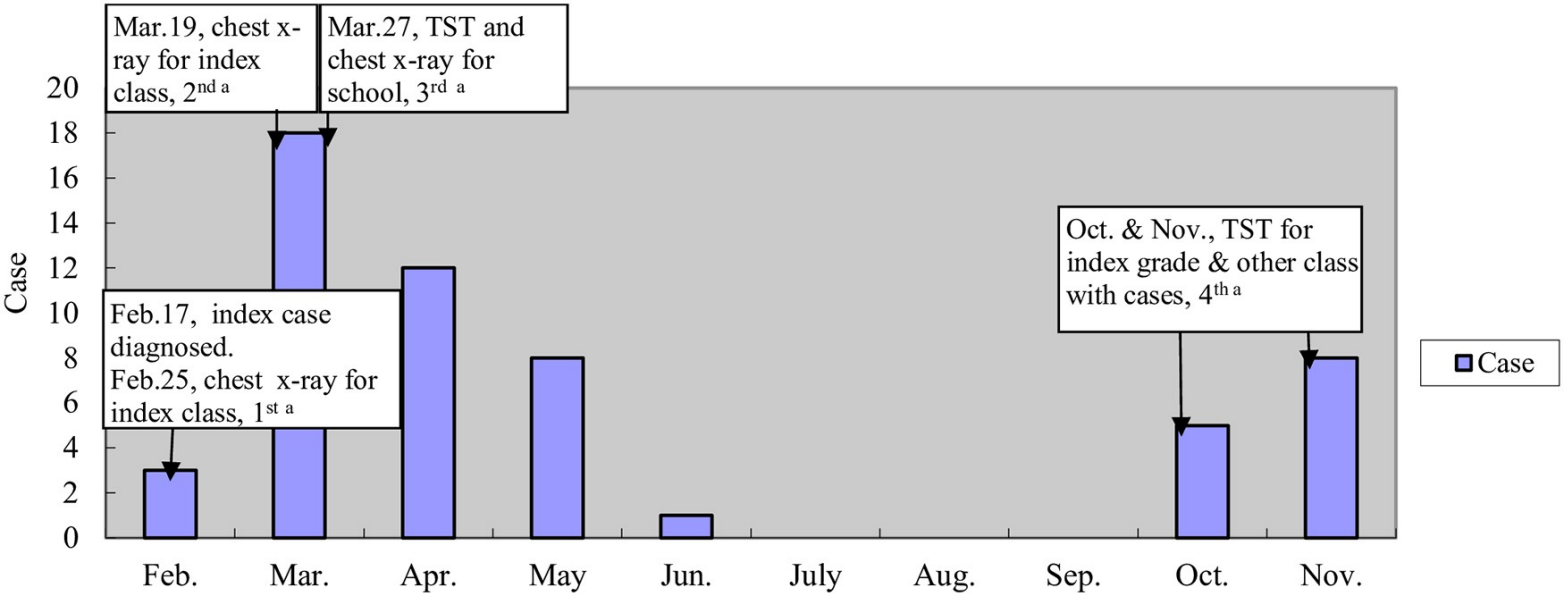
Timelines of TB screening and of cases diagnosed. ^a^ The four TB screenings. Abbreviations: TB, tuberculosis; TST, tuberculin skin test.

Following the diagnosis of the index case, four TB screenings were carried out in the school (Fig 1). Except for cases found in the screenings, there were also cases diagnosed through clinical consultation of students with TB-suspicious symptoms between the screening intervals.

1. The first two screens. Because of the shortage of tuberculin in China in 2013, the first two screenings were conducted by Jingli Hospital, a large-scale private general-hospital, using only chest x-ray for the index class.
2. For the third screening, interview of TB-suspicious symptoms and TST for junior students, and chest x-ray for senior students and staff, were launched. Those with TB-suspicious symptoms or a SMP or abnormal chest x-ray were further screened by chest digital radiography (DR). Chest x-ray was carried out by Hebi CDC and Jingli hospital, and chest DR, by Hebi Contagious Disease Hospital.
3. In the fourth screening, TST was given to students without TB diagnosed and with TST indurations less than 15mm in the third screening (to find those who had been infected but were at ‘window period’ of TST reaction at the time of first TST) for the index grade and other classes which have had TB cases diagnosed. Chest radiography was taken for those with SMP in the fourth or the third screening, as well as for all students without TB diagnosed in the index class no matter their TST results. It was carried out by Hebi Contagious Disease Hospital.

All subjects with clinical or radiography findings suggestive of TB submitted three sputum specimens for smear microscopy, and were given a computed tomography (CT) scan or diagnostic therapy, if necessary. Sputum samples from parts of TB cases were provided for liquid culture. SS microscopy and diagnostic treatment with antibiotics was carried out by Hebi Contagious Disease Hospital. SC and mycobacterial interspersed repetitive unit-variable number tandem repeat (MIRU-VNTR) genotyping on 24 loci for culture-confirmed TB were conducted and analyzed in the TB reference laboratory at the Henan CDC, according to their standard operating procedures. Diagnosis of all the TB cases, including those found through clinical consultation, was co-made by the clinicians from the Hebi Contagious Disease Hospital and the Henan CDC. The last two screenings were supervised by experts from the Henan CDC.

### Outbreak investigation

The index case was a female boarder, in senior grade two. She developed a productive cough in October 2012, and sought medical care repeatedly in private clinics when her symptoms worsened in December 2012, but was misdiagnosed and given antibiotic treatment. She was hospitalized after the winter vacation and diagnosed as SS positive PTB on February 17, 2013. She had attended school during the 4 months from the onset of her symptoms until the winter vacation.

On February 25, 2013, the first screening of all 78 students (excluding the index case) in the index class and 8 teachers who shared the classroom with the index case was launched. Three tuberculous pleurisy cases were diagnosed among the students. Subsequently, three additional students in the class were diagnosed as TB through clinical consultation. The second screening was conducted for 69 students in the same class (seven cases were already diagnosed and three others were seeking clinical consultation because of having symptoms) on Mar. 19, 2013. Thirteen additional TB cases were detected (one was SS positive) in the index class from Mar.19 to 23, 2013. Another TB case in senior grade one, who was hospitalized from Nov. 2012, was also identified in clarifying the status of students in the school during this period. The third TB screening of the school students and staff was carried out from Mar. 27 to 30, 2013, and fifteen TB cases were diagnosed in students and none in staff. Another six students were diagnosed with TB through for clinical consultation from Apr. 20 to June 15, 2013. Then, another thirteen TB cases were detected by the fourth screening from November to December 2013 (Fig). In total, 55 TB cases were detected in the school from February to November 2013. Case investigation was carried out for all of them, and the results showed none of them had a clear contact history of a TB patient outside school or at home.

Liquid SC was done for sputum samples of 30 cases diagnosed from March 23 to April 18, 2013. Nine cases were SC positive (1 SS positive, 8 SS negative), and all were the classmates of the index case. All the nine mycobacterium TB isolates from the nine students with culture-confirmed TB, analyzed by mycobacterial interspersed repetitive unit-variable number tandem repeat (MIRU-VNTR) genotyping on 24 loci, revealed that all the nine strains were identical, except for the failure of amplication on VNTR42 loci for one strain, and a different repeat number on QUB26 loci for another strain with the other 8 strains (see S1 Table).

### Statistical analysis

Logistic regression models were implemented to examine the association between TB disease, SMP and potential risk factors, and to estimate odds ratios (OR) and 95% confidence intervals (CI), using Stata (version 11.0, Stata Corporation, College Station, Texas, USA). Risk factors included gender, classroom and residential status of the students. To stratify contact levels of students with the index case, we categorized classroom status as same classroom (with the index case), same floor but different classrooms, same teaching-building but different floors, and different teaching-buildings, and residential status as same dorm-floor, different dorm-floors in same building, different dorm-buildings, and commuting. Initial unadjusted analyses were performed for all potential risk factors to identify those significantly associated with SMP and TB disease. Comparisons between categorical variables were analyzed using *chi square* test or Fisher’s exact test, as appropriate. All statistical tests were two sided and P<0.05 was considered statistically significant. To identify factors independently associated with the outcome variable, we performed a multiple logistic regression analysis. Logistic regression models were also used to examine the association between TB disease and different TST indurations.

### Ethical considerations

This investigation was a response to a public health emergency and was exempted by the Institutional Review Board (IRB) of the Henan CDC. To protect the confidentiality of the participants, all data were obtained in a fully anonymized and de-identified manner, and received the approval from the IRB of Henan CDC.

## Results

### SMP in the third screening

Among 4102 students and 289 staff in the school, 3996 (97.42%) and 278 (96.19%) had TST results in the third screening (from March 27 to 30, 2013), respectively. About 3.55% (142/3996) students and 16.55% (46/278) staff had SMP. The SMP rate in staff was significantly greater than that in students (*x*^2^ = 104.35, p<0.001). 20.29% of students in the index class and 3.26% in the other classes were SMP. The SMP rate in the index class was significantly higher than that of the other classes (p<0.001) (Table 1).

**Table 1:** SMP and TB cases of students in different classes.

	N of screened	n	%	*p* value ^b^
**SMP** ^a^				
Index class	69	14	20.29	<0.001
Non-index classes	3927	128	3.26	
**TB cases**				
Index class	79	33	41.77	<0.001
Non-index classes	4003	22	0.55	

^a^ TST (tuberculin skin test) results in the 3rd screening (Mar.27–30, 2013)

^b^ Fisher exact

Abbreviations: N (n), number; SMP, strong Mantoux positive; TB, tuberculosis

### TB cases diagnosed

From February to November 2013, 4082 students and 278 staff were screened (symptoms interview, TST and/or chest x-ray) in the four screenings or went to see a doctor for TB suspicious symptoms (including the index case), and 55 TB cases in students and none in staff were diagnosed, with an incidence of 1.35% for students. Out of the 55 TB cases, ten (18.18%) were laboratory-confirmed and the other 45 (81.82%) were clinically diagnosed. Ten were tuberculous pleurisy and 45 were pulmonary TB (out of whom, two were complicated with pleurisy). 34 (61.82%) of the 55 cases were with symptoms. 41 (74.55%) of them were boarding students and 14 (25.45%) were commuting ones. Out of the 79 students in the index class, 33 (5 males, 28 females) were diagnosed as TB cases, with a detection rate of 41.77% (27.78% for males, 45.9% for females). The other 22 cases were distributed in other thirteen classes involving four grades, with a detection rate of 0.55%. The TB detection rate in the index class was significantly higher than that in the other classes (p<0.001) (Table 1). Out of the 40 boarders in the index class who lived in the same or neighboring four dormitories with the index case, nineteen (47.50%) were diagnosed as TB cases.

### SMP and TB cases detected for students in different residential status

Out of the 2272 boarding students with TST results in the third screening, 95 (4.18%) were SMP. While, 47 (2.73%) out of the 1724 commuters were SMP. The SMP rate in the boarders was significantly higher than that in the commuters (p<0.05). Case detection rate in the boarders (1.77%) was also significantly higher than that in the commuters (p<0.01) (Table 2).

**Table 2:** SMP and TB cases of students in different residential status.

Residential state	SMP^a^					TB Cases				
	N	n	%	*x*^2^	p value	N	n	%	*x*^2^	p value
Boarders	2272	95	4.18	6.06	0.014	2319	41	1.77	7.15	0.008
Commuters	1724	47	2.73			1763	14	0.79		
**Total**	3996	142	3.55			4082	55	1.35		

^a^ TST (tuberculin skin test) results in the 3rd screening (Mar.27–30, 2013)

Abbreviations: N (n), number; SMP, strong Mantoux positive; TB, tuberculosis

### TB cases in different TST indurations and different classes

Out of the 55 TB cases diagnosed, 41 had TST results in the third screening. Among the other fourteen cases without TST results, eight were diagnosed with TB before the screening and six were for other reasons. The TB detection rate was 0.11%, 0.88%, 10.99% and 9.15% in students with TST indurations of 0mm-, 5mm-, 10mm- and ≥15mm, respectively. Compared with those with TST indurations of 0mm-, ORs of TB cases were 7.94 (95%CI: 2.14–29.37, p = 0.002), 101.14 (95%CI: 29.13–351.16, p<0.001) and 89.82 (95%CI: 25.28–319.13, p<0.001) for that of 5mm-, 10mm- and ≥15mm, respectively (Table 3).

**Table 3:** TB case detection rate in different TST indurations.

TST ^a^		TB Cases ^b^				
Induration (mm)	N	n	%	OR	95% CI	P value
0-	2677	3	0.11	Reference		
5-	1020	9	0.88	7.94	2.14,29.37	0.002
10-	157	16	10.19	101.14	29.13,351.16	<0.001
≥15	142	13	9.15	89.82	25.28,319.13	<0.001

^a^ TST results in the 3rd screening (Mar.27–30, 2013)

^b^ Of the 55 cases diagnosed, 14 without TST results (8 for diagnosed before the screening, 6 for other reasons)

Abbreviations: N (n), number; TST, tuberculin skin test; OR, odds ratio; CI, confidence interval; TB, tuberculosis

In the index class, the TB detection rate in students with SMP for the third screening was not significantly different with that in those with TST <15mm (21.43% vs 38.18%; p>0.05). However, it was significantly higher in non-index classes (7.81% in students with SMP vs 0.18% in those with TST <15mm) (Table 4).

**Table 4:** Detection rates of TB cases with different TST indurations in different classes.

TST ^a^		Index class		Non-index classes		
Indurations (mm)	N	TB cases ^b^ (%)	*p* value ^c^	N	TB cases ^b^ (%)	*p* value ^c^
<15	55	21 (38.18)	0.3496	3799	7 (0.18)	<0.001
≥15	14	3 (21.43)		128	10 (7.81)	

^a^ TST results in the third screening (Mar.27–30, 2013)

^b^ Of the 55 cases diagnosed, 41with TST results in the third screening

^c^ Fisher exact

Abbreviations: TST, tuberculin skin test; N, number

### Case finding and TST results at the fourth screening

Thirteen TB cases in students were detected at the fourth screening. Out of them, four were diagnosed in those with TST indurations ≥15mm and one other was without TST result at the third screening. 760 students with TST indurations <15mm at the third screening were screened at the fourth one, and 68 (8.95%) of them converted to ≥15mm and eight (11.76%) cases were diagnosed out of the converted ones.

### Unadjusted analysis: Risk factors for SMP and TB cases

Univariate logistic regression models were used to explore the association between characteristics of students with SMP (Table 5) and TB cases (Table 6), respectively. Compared with males, commuting students and those in different teaching-buildings with the index case, respectively, females; sharing same dormitory-floor, different floor in same dormitory-building; and same classroom, different classroom on same teaching-floor, different floors in same teaching-building were all significantly associated with the risk of both SMP and TB disease. The ORs of all these risk factors for SMP and TB cases were at least 2, except for those for SMP of a different floor in the same dormitory-building (OR 1.88), and a different floor in the same teaching-building (OR 1.87). On the other hand, the univariate logistic models did not show significant associations between SMP or TB disease and resided in different dormitory-buildings from the index case. The closer the contact with the index case, the larger the ORs for both SMP and TB disease observed in the univariate logistic regression models.

**Table 5:** Distribution, unadjusted OR and 95% CI for characteristics of students on SMP.

Characteristics	N of TST	SMP				
		n	%	OR	95%CI	p value
**Gender**						
Female	2137	101	4.73	2.20	1.52, 3.18	<0.001
Male	1859	41	2.21	Reference		
**Residential** ^a^						
Same dorm-floor	162	29	17.90	8.08	4.90, 13.30	<0.001
Different floor, same dorm-building	557	27	4.85	1.88	1.15, 3.05	0.011
Different dorm-building	1553	39	2.51	1.00	0.65, 1.53	0.995
Commuting	1724	47	2.73	Reference		
**Classroom** ^a^						
Same classroom	69	14	20.29	9.73	5.20, 18.20	<0.001
Different classroom, same floor	152	19	12.5	5.46	3.22, 9.26	<0.001
Different floor, Same teach-building	599	28	4.67	1.87	1.21, 2.91	0.005
Different teach-building	3176	81	2.55	Reference		

^a^ Association with the index case

Abbreviations: TST, Tuberculin skin test; SMP, strong Mantoux positive; N(n), Number; OR, Odds ratio; CI, Confidence interval

**Table 6:** Distribution, unadjusted OR and 95% CI for characteristics of students on TB cases.

Characteristics	N of screened	TB cases				
		n	%	OR	95%CI	*p* value
**Gender**						
Female	2191	45	2.05	3.94	1.98, 7.85	<0.001
Male	1891	10	0.53	Reference		
**Residential** ^a^						
Same dorm-floor	171	23	13.45	20.76	10.31, 41.84	<0.001
Different floor, same dorm-building	573	11	1.92	2.60	1.16, 5.84	0.020
Different dorm-building	1575	7	0.44	0.68	0.28, 1.64	0.388
Commuting	1763	14	0.79	Reference		
**Classroom** ^a^						
Same classroom	79	33	41.77	256.35	116.06, 566.21	<0.001
Different classroom, same floor	155	7	4.52	16.90	6.21, 46.00	<0.001
Different floor, Same teach-building	623	6	0.96	3.47	1.23, 9.80	0.019
Different teach-building	3225	9	0.28	Reference		

^a^ Association with the index case

Abbreviations: N(n), number; TB, tuberculosis; OR, Odds ratio; CI, Confidence interval

### Adjusted analysis: Risk factors for SMP and TB disease

Factors significantly associated with risk of SMP and TB disease in students were, sharing same dormitory-floor (AOR 5.36, 95% CI 3.02–9.48, p<0.001 for SMP; AOR 2.67; 95% CI 1.03–6.96, p = 0.044 for TB cases), same classroom (AOR 4.01; 95% CI 1.95–8.26, p<0.001 for SMP; AOR 165.08; 95% CI 66.88–407.47, p<0.001 for TB cases), same teaching-floor in different classroom (AOR 5.41; 95% CI 3.02–9.71, p<0.001 for SMP; AOR 11.24; 95% CI 3.71–34.03, p<0.001 for TB cases) and same teaching-building on different floor (AOR 1.81; 95% CI 1.16–2.85, p = 0.009 for SMP; AOR 3.30; 95% CI 1.16–9.42, p = 0.025 for TB cases). For classroom status in this model, the closer the contact with the index case, the larger the AORs for SMP and TB disease observed in these models. Gender, accommodated on different floors of the same dormitory-building or in a different dormitory-building from the index case were not significantly associated with SMP or TB disease (Table 7).

**Table 7:** Adjusted OR and 95% CI for characteristics of students on SMP and TB cases.

Characteristics	SMP			TB Case		
	AOR	95% CI	P value	AOR	95%CI	P value
**Gender**						
Female	1.5	0.96, 2.34	0.075	1.36	0.48, 3.85	0.559
Male	Reference			Reference		
**Residential** ^a^						
Same dorm-floor	5.36	3.02, 9.48	<0.001	2.67	1.03, 6.96	0.044
Different floor, same dorm-building	1.13	0.65, 1.96	0.662	2.07	0.75, 5.68	0.158
Different dorm-building	1.2	0.77, 1.88	0.418	1.02	0.35, 2.97	0.970
Commuting	Reference			Reference		
**Classroom** ^a^						
Same classroom	4.01	1.95,8.26	<0.001	165.08	66.88, 407.47	<0.001
Different classroom, same floor	5.41	3.02, 9.71	<0.001	11.24	3.71, 34.03	<0.001
Different floor, Same teach-building	1.81	1.16, 2.85	0.009	3.30	1.16, 9.42	0.025
Different teach-building	Reference			Reference		

^a^ Association with the index case

Abbreviations: SMP, strong Mantoux positive; TB, tuberculosis; AOR, Adjust odds ratio; CI, Confidence interval

## Discussion

TB outbreaks usually occur in people who live in a closed and crowded environment. TB outbreaks have occurred in refugees [10–12], correctional facility [13], long-term care facility [14], schools [15,16] and on a school bus [17] in other countries. TB outbreaks in schools have frequently been reported in China [2, 6, 18–20]. The special environment in schools in which shared living space and close proximity facilitates the outbreaks of TB [5]. Contributing factors include sustained contact, inadequate classroom ventilation, and delayed diagnosis [5, 18]. All of these factors existed in our study. First, the index case was a female boarder and she developed a productive cough in October 2012, and sought medical care repeatedly in private clinics when her symptoms worsened in December 2012. She was not diagnosed with TB until she was hospitalized after winter vacation and diagnosed as sputum smear positive TB on February 17, 2013. Consulting and diagnostic delay for her lasted for 4 months, during which she attended and lived at the school, continuing close contact with her classmates. Second, it was winter and cold during the 4 months. There was no mechanical ventilation, and windows were usually closed in the classrooms and dormitories [2, 6], which facilitated the spread of tubercle bacilli in this school. Diagnostic delay is a primary contributing factor of TB outbreaks, and delay is usually due to not recognizing TB symptoms or other diagnostic errors [5]. It reminds us that it is urgent to disseminate knowledge about TB among medical staff and the public, especially among students, to improve their awareness of TB and reduce the delay seeking care and diagnosis.

We retrospectively reviewed the TB outbreak data which occurred in the cohort of students and staff of the school from February to November 2013 to identify factors related to the detection rate of TB disease and SMP in school settings. Students in middle schools in China usually have limited contact with the general population because of their high burden of study. They have fixed classrooms, and fixed dormitories for boarding students. Public places, such as libraries in schools, are rarely visited by them whereas the dining hall is frequently used by boarding students. Because students in the same grade are usually the same age, we only included gender, residence and classroom status of the students as risk factors for SMP and TB cases in our analysis.

The results of the univariate analysis in our study showed that the following characteristics of students were all related to both SMP and TB disease: female, same dorm-floor, different floor in same dorm-building, and same classroom, different classroom in same teaching-floor, different floor in same teaching-building with the index case. These were consistent with what Fang reported that the same classroom with the index case was the primary risk factor for SMP and disease [18]. However, our study identified additional risk factors. Results of the multivariate analysis in our study showed that sharing the same dorm-floor; the same classroom, different classrooms in same teaching-floor, and different floors in same teaching-building with the index case were independent risk factors both for SMP and TB disease. Gender and different floor in same dorm-building were no longer associated with either SMP or TB disease. The closer the contact was with the index case, the larger the adjusted OR for SMP and TB disease observed in these models, which reminds us that the risk of infection and developing disease was associated with the level of exposure, which is in accordance with what Rodríguez [5] and Lu [19] reported. It also reminds us that we should not only focus on screening the index class when dealing with a TB outbreak in a school, but also focus on the same dorm-floor with the index case, and that we should expand the screening gradually to the same teaching-floor and same teaching-building based on the epidemics.

The result showed that the detection rate for both TB case and SMP was much higher for females than that for males in the univariable analysis, even if the dining hall was frequently used by boarding students, and all of them, no matter what gender they were, shared the same dining hall, The reasons were as following. First of all, the index case was a female, boarding student, and 61 (77.2%) of the total 79 students in her classroom were females. It was clear that most of the closer contacts for the index case were female students in the same class, same dormitory, or even same dorm-floor, which resulted in the detection rate higher in female for boarding students. Secondly, compared with the students in the same classroom, same dorm-floor, the frequency and period contacted with the index case were relatively less for those contacted in the dining hall, which resulted in a relatively lower detection rate for those contacts in the dining hall, even if they had the same exposure opportunity for males and females. After multivariable analysis, there was no statistical significance between genders, suggesting that gender was a confounding factor in this study.

In our study, TB cases were diagnosed in students with any TST induration at the third screening. Generally, the larger the TST induration, the larger the risk of TB diseases observed, except for that TB detection rate in TST indurations of 10mm- (10.99%) was greater than that ≥15mm (9.15%). Further analysis for different classes discovered that the TB detection rate in students with SMP was not significantly higher than that <15mm in the index class but was significantly higher in the other classes (7.81% in students with SMP vs 0.18% in those with that <15mm). It reminds us that the risk to develop disease is relatively higher after a long period of close contact with the index case, no matter what the TST indurations are. Further and cohort studies are needed for the correlation of TB with different TST indurations after an outbreak.

In China, screening of contacts is a requirement for TB outbreak investigations and control in a school, and TST is usually carried out as one of the screening methods. And a repeated TST is obligatory for those without SMP in a TB outbreak in school by the norm of TB control in school [6]. Usually, more attention were paid to contacts with SMP compared to those with TST indurations less than 15mm, because a TST indurations of 15mm or larger and /or blisters, necrosis or lymphangitis are criteria of latent TB infection (LTBI) and for prophylaxis [6], as well as one of the supplementary criteria for diagnosis of TB cases in China [7]. From this study, however, we learned that a fairly large number (21/55) of the students with TST less than 15mm in the index class developed TB during the study period whereas only 0.18% (7/3799) of those in the other classes were diagnosed as TB cases. It reminds us that we should also pay attention to contacts with TST less than 15mm in the index class and provide them with following-up service for at least one year (because of the high risk from infection to illness during the first two years), in addition to those with SMP.

13 cases were diagnosed at the 4^th^ screening after the dealing of outbreak. Out of them, eight were in index class and five in other classes. These results remind us that the risk of TB is very high after a long period of close contact with a contagious case, and that it was important that another screening was carried out 3 months after the outbreak, especially for the index class.

Rodríguez [5] and colleagues’ analysis of outbreaks in nurseries confirmed the speed and ease with which the infection progresses to disease in preschool children, with a very high risk of developing disease in infected children irrespective of the level of contact. We also learned from our study how fast and easy it was for infection to progress to disease even in high school, especially in the index class; and that there still were cases diagnosed one after another and at the screening 6 months later despite launching extensive TB screening in students.

This study had certain limitations. First, TST was not conducted in a portion of cases (14 cases) due to a shortage of PPD and were diagnosed before the TST screening or other reasons. This might result in an underestimation of SMP and the odds ratios of risk factors for SMP and may result in bias in analyzing the odds ratio of the TB detection rate from different TST indurations. Second, our preliminary study to the relation of TB disease and TST indurations was based on the only TST result of the third screening for students. The lacking of any previous TST information for students might affect the accuracy of the relation, because we could not exclude the possibility of the previous infection before this outbreak. Thirdly, our recommendation for screening to deal with a TB outbreak may fit only to middle schools because students in universities usually have more close contact populations. Fourthly, only 30 sputum samples were cultured because the local CDC and contagious disease hospital could not perform sputum cultures, which might result in the underestimation of laboratory-confirmed cases. Lastly, we could not confirm that the linkage of all the TB patients even if all the nine isolates from the nine students were identical, analyzed by MIRU-VNTR genotyping techniques because of the limited culture positive sputa, and we could not completely exclude the probability of infection source outside school even if the results of case investigation showed none of them had a clear contact history of a TB patient outside school or at home, and the detection rates of SMP and TB cases among commuters were significantly lower than that among boarders.

## Conclusion

Same dormitory-floor, same classroom, same teaching-floor and same teaching-building with the index case were all risk factors for both TB infection and disease for students in the outbreak, and the closer the contact was with the index case, the larger the risk observed. Attention should also be paid to students with TST indurations <15mm, as well as to those with indurations ≥15mm in the index class in dealing with the outbreak.

## Supporting information

S1 Table (DOCX)

S1 File Dataset. (CSV)
