# Supplementary material for: An outbreak of tuberculosis in a middle school in Henan, China: Epidemiology and risk factors
Source: PLoS One. 2019 Nov 15;14(11):e0225042. doi: 10.1371/journal.pone.0225042 (PMC6857903; doi:10.1371/journal.pone.0225042)
Supplement: S1 Table — (DOCX) [file pone.0225042.s001.docx]

S1.Table

Table MIRU-VNTR Profiles of all the 9 mycobacterium tuberculosis isolates from the 9 students

| Strains | Mtub21 | ETR-A | MIRU02 | MIRU04 | MIRU10 | MIRU16 | MIRU20 | MIRU23 | MIRU24 | MIRU26 | MIRU27 | MIRU39 | MIRU31 | MIRU40 | QUB26 | QUB-11b | VNTR43 | VNTR46 | VNTR48 | VNTR49 | VNTR52 | VNTR53 | VNTR42 | VNTR47 |
| --- | --- | --- | --- | --- | --- | --- | --- | --- | --- | --- | --- | --- | --- | --- | --- | --- | --- | --- | --- | --- | --- | --- | --- | --- |
| M-10 | 6 | 4 | 2 | 2 | 3 | 3 | 2 | 5 | 1 | 7 | 3 | 3 | 5 | 2 | 8 | 6 | 4 | 4 | 2 | 3 | 3 | 2 | 4 | 4 |
| M-11 | 6 | 4 | 2 | 2 | 3 | 3 | 2 | 5 | 1 | 7 | 3 | 3 | 5 | 2 | 8 | 6 | 4 | 4 | 2 | 3 | 3 | 2 | 4 | 4 |
| M-12 | 6 | 4 | 2 | 2 | 3 | 3 | 2 | 5 | 1 | 7 | 3 | 3 | 5 | 2 | 8 | 6 | 4 | 4 | 2 | 3 | 3 | 2 | 4 | 4 |
| M-15 | 6 | 4 | 2 | 2 | 3 | 3 | 2 | 5 | 1 | 7 | 3 | 3 | 5 | 2 | 8 | 6 | 4 | 4 | 2 | 3 | 3 | 2 | 4 | 4 |
| M-17 | 6 | 4 | 2 | 2 | 3 | 3 | 2 | 5 | 1 | 7 | 3 | 3 | 5 | 2 | 8 | 6 | 4 | 4 | 2 | 3 | 3 | 2 | 4 | 4 |
| M-22 | 6 | 4 | 2 | 2 | 3 | 3 | 2 | 5 | 1 | 7 | 3 | 3 | 5 | 2 | 7 | 6 | 4 | 4 | 2 | 3 | 3 | 2 | 4 | 4 |
| M-23 | 6 | 4 | 2 | 2 | 3 | 3 | 2 | 5 | 1 | 7 | 3 | 3 | 5 | 2 | 8 | 6 | 4 | 4 | 2 | 3 | 3 | 2 | 4 | 4 |
| M-5 | 6 | 4 | 2 | 2 | 3 | 3 | 2 | 5 | 1 | 7 | 3 | 3 | 5 | 2 | 8 | 6 | 4 | 4 | 2 | 3 | 3 | 2 | 4 | 4 |
| M-9 | 6 | 4 | 2 | 2 | 3 | 3 | 2 | 5 | 1 | 7 | 3 | 3 | 5 | 2 | 8 | 6 | 4 | 4 | 2 | 3 | 3 | 2 |  | 4 |
